# Enhancement of Photocurrent Stability in Oxidation-Resistant WSe_1.95_Te_0.05_ Nanosheets

**DOI:** 10.1021/acsomega.5c02983

**Published:** 2025-06-13

**Authors:** Shiu-Ming Huang, Ankush Saxena, Pin-Cing Wang, Tzu-Yueh Tu, Ruei-San Chen, Mitch M. C. Chou

**Affiliations:** † Department of Physics, 34874 National Sun Yat-Sen University, Kaohsiung 804, Taiwan (R.O.C.); ‡ Program on Key Materials, Academy of Innovative Semiconductor and Sustainable Manufacturing, 34912 National Cheng-Kung University, Tainan 70101, Taiwan (R.O.C.); § Graduate Institute of Applied Science and Technology, 34878 National Taiwan University of Science and Technology, Taipei 10607, Taiwan (R.O.C.)

## Abstract

The electrical and optical characteristics of semimetallic WSe_1.95_Te_0.05_ nanosheets have been comprehensively studied. The current–voltage measurements reveal a linear response when they are interfaced with platinum (Pt) electrodes, confirming the establishment of an ohmic contact between the WSe_1.95_Te_0.05_ nanosheets and the Pt contacts. Additionally, the observed photocurrent demonstrates a direct linear correlation with the applied voltage and scales proportionally with the intensity of the incident light. Remarkably, the device achieves an exceptional photoresponsivity of 108 A/W at 0.5 V without requiring surface modifications. Furthermore, the electrical resistivity and photocurrent exhibit admirable stability, remaining unaffected by air exposure under ambient conditions for more than six months. These results underscore the inherent capabilities of WSe_1.95_Te_0.05_ nanosheets and their potential as highly reliable materials for advanced photodetection applications.

## Graphical abstract

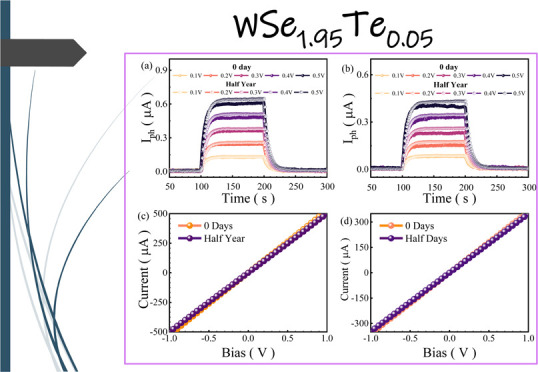

## Introduction

High photocurrent response systems are vital for photosensor applications.^1^ Nanosheets are particularly advantageous due to their minimal light penetration depth, allowing for efficient photoresponse optimization. While external geometric modifications can enhance performance, the intrinsic band structure fundamentally governs the photoresponse and carrier transport. Low-dimensional (LD) materials, such as graphene, graphene-based heterostructures, and topological insulators (TIs), exhibit unique properties such as linear energy-momentum relations. These materials offer fast response times, broad spectral sensitivity, high responsivity, and exceptional detectivity, making them suitable for diverse applications.^2–5^ 2D transition metal dichalcogenides (TMDs) are promising for next-generation electronics and optoelectronics due to their distinctive band structures and adjustable bandgaps.^6–8^ However, their high surface-to-volume ratios make them susceptible to oxidation, which degrades the photocurrent performance and limits their utility in electronic devices. Monolayer materials such as MoS_2_, WS_2_, and WSe_2_, have gained significant attention for their exceptional optoelectronic properties. Due to their atomically thin structure and direct bandgaps, these 2D materials exhibit strong light–matter interactions, making them highly suitable for photodetector applications. Among them, monolayer MoS_2_ stands out with photoresponsivity values exceeding 10^3^ A/W under certain conditions,^9^ owing to efficient carrier trapping mechanisms and long photocarrier lifetimes. Some hybrid structures or engineered heterojunctions based on monolayers have reported even higher responsivities, reaching up to 10^6^ A/W,^10^ positioning them among the most sensitive light detectors at the nanoscale. In the family of TMDs, WSe_2_ has garnered significant attention due to its executive electrical and optical properties, such as higher photocurrent response. It is a layered van der Waals material that has an ∼1.6 eV direct bandgap in monolayer and ∼1.2 eV in bulk.^11–14^ It has both types of charge carriers, which depend on gate bias or doping conditions with a higher on/off current ratio (>10^6^).^15^ The Pt electrodes play a vital role in influencing the photocurrent response of WSe_2_ by either contact resistance or carrier dynamics. The combination of Pt electrodes and Te doping in WSe_2_-based devices significantly enhances the photocurrent response by improving both charge injection and carrier dynamics. Pt electrodes, with their favorable work function (∼5.6 eV), form a Schottky or near-Ohmic contact with WSe_2_, facilitating efficient hole injection, reducing contact resistance, and enhancing the overall photocurrent.^16–18^ The addition of Te doping further optimizes this performance by narrowing the bandgap of WSe_2_. The doping broadens the optical absorption spectrum and shifts the absorption edge toward lower energies, enabling better sensitivity to infrared light. Additionally, Te doping introduces extra electronic states, reducing the Schottky barrier height at the Pt-WSe_2_ interface and improving charge injection and carrier mobility.^19^ This combination of Pt electrodes and Te doping leads to enhanced carrier transport, increased photocurrent density, and higher responsivity, making the material more effective for a wide range of optoelectronic applications, including photodetectors and solar cells. In this work, we explore the photocurrent response of WSe_1.95_Te_0.05_ nanosheets. These nanosheets display a linear current–voltage relationship with Pt electrodes, confirming the formation of an ohmic contact. The photocurrent scales linearly with the applied voltage and incident light intensity, achieving a photoresponsivity of 108 A/W at 0.5 V under 532 nm illumination.

## Experimental Method

Tellurium-doped tungsten diselenide (WSe_1.95_Te_0.05_) single crystals were synthesized by using the chemical vapor transport (CVT) method. High-purity tungsten powder (99.99%), selenium, and tellurium were sealed in a silica ampule and evacuated to 10^–3^ Torr. The synthesis involved three stages: first, the raw materials were heated to 600 °C over 95 h to form polycrystalline powder. Next, the material was annealed at 1050 °C for 96 h. Finally, the annealed powder was sealed in a 20 cm silica tube, placed in a two-zone furnace, heated to 1020 °C, and gradually cooled to 980 °C over 170 h. The crystals were then furnace-cooled to room temperature. The resulting crystals, cleaved along the basal plane, revealed a silvery reflective surface and were prepared for characterization. WSe_1.95_Te_0.05_ nanosheets were synthesized and characterized using a range of structural, morphological, and electrical techniques for two samples, i.e., Sample-S1 and Sample-S2. The crystallinity and phase purity were confirmed through single-crystal X-ray diffraction (SCXRD) using a Bruker D8 Venture system. A selected area electron diffraction (SAED) experiment is performed using a transmission electron microscope (TEM) equipped with a selected area aperture to isolate a hexagonal structure of the sample. Raman spectroscopy (Renishaw inVia or Horiba LabRAM HR Evolution) was employed to investigate vibrational modes and assess the impact of Te incorporation into the WSe_2_ lattice. The surface morphology and thickness of the nanosheets were examined using scanning electron microscopy (SEM) (FEI Nova NanoSEM or Zeiss Sigma 300) and atomic force microscopy (AFM) (Bruker Dimension Icon). Electrical transport properties were evaluated through *I–V* measurements conducted with a Keithley 2400 source meter. To assess long-term stability, time-dependent current measurements were performed immediately after fabrication and again after six months using a Keithley 2450 source meter under continuous illumination from a calibrated light source (Newport Xenon Lamp). Additionally, the responsivity as a function of incident power was measured using a Newport power meter with a controlled laser source, providing insights into the optoelectronic performance of the nanosheets. X-ray photoelectron spectroscopy (XPS) measurements were performed using a monochromatic Al Kα source (1486.6 eV) under ultrahigh vacuum (∼10^–9^ mbar). A hemispherical analyzer was employed to record the core-level spectra with an energy resolution of ∼0.5 eV. The studies were done within different conditions such as immediately after fabrications and after six months. During the six months of waiting period, the sample was stored in a normal chamber without any special treatment.

## Results and Discussion

The single-crystal X-ray diffraction (SCXRD) of WSe_1.95_Te_0.05_ is shown in Figure 1a. The six peaks are observed in the SCXRD that aligns with standard reference data (JCPDS 38–1388), which confirms the phase purity of as-grown WSe_1.95_Te_0.05_. The full width at half-maximum (fwhm) of the peak (002) is found to be 0.14°, elucidating the phase purity of the crystal. The high-resolution transmission electron microscopy (HRTEM) pattern is shown in the inset of Figure 1a. The image reveals a well-ordered atomic structure at a 1 nm scale with distinct diffraction spots arranged in a hexagonal pattern, indicating the crystalline nature of the material. The red-circled spots highlight the periodic atomic arrangement, suggesting a high degree of structural order. The measured interatomic distance of 6.8 Å, as indicated in the figure, provides essential information about the lattice spacing, which can be used for material identification by comparing it with known crystallographic data. The hexagonal symmetry suggests that the material could belong to a class of hexagonal close-packed (HCP) structures or layered two-dimensional (2D) materials such as graphene, MoS_2_, or hexagonal boron nitride (h-BN). The observed structural periodicity and crystallographic details provide valuable insights into the material’s atomic arrangement, which is critical for understanding its properties and potential applications in nanotechnology and electronic devices. The Raman spectrum of WSe_1.95_Te_0.05_, as shown in Figure 1b, provides critical insights into the vibrational properties and structural modifications induced by Te substitution in the WSe_2_ lattice. The experimental Raman intensity is in a well-matched fitting, which corresponds to the cumulative peak fit. The Lorentz fitted Raman spectrum elucidates the several phonon modes, which are deconvoluted and labeled according to their respective contributions to the overall spectrum. The prominent peaks at 250 cm^–1^ correspond to the characteristic A_1g_ and E_2g_^1^ modes,^20^ which are intrinsic to the layered hexagonal structure of tungsten diselenide (WSe_2_). The A_1g_ mode represents out-of-plane vibrations of the chalcogen atoms, while the E_2g_^1^ mode corresponds to in-plane vibrations.^21^ The presence of these peaks confirms the retention of the fundamental structural integrity of WSe_2_ despite minor Te incorporation. However, a slight broadening and shift of these peaks indicate subtle lattice distortions and increased phonon scattering due to Te substitution. In addition to the fundamental modes, several combinations and difference modes are observed, reflecting multiphonon interactions in the system. The low-frequency peaks below 100 cm^–1^ correspond to the longitudinal acoustic (LA) phonon mode and its combination with optical modes, such as A_1g_ – LA­(M) and A_2g_ – LA­(M). These modes arise due to phonon–phonon interactions and interlayer coupling effects, which are typically prominent in layered transition metal dichalcogenides (TMDs).^22^ The presence of these acoustic modes further indicates the high crystalline quality of the sample and the preservation of the interlayer interactions. The A_1g_ + LA­(M) mode appearing at an intermediate frequency signifies an interaction between the out-of-plane optical mode and the longitudinal acoustic mode, suggesting that Te incorporation alters the interatomic force constants slightly. The broadening of these modes and minor frequency shifts relative to those of pristine WSe_2_ suggest the influence of strain, alloy disorder, and mass contrast between Se and Te atoms. The introduction of Te introduces local distortions in the lattice, slightly perturbing the phonon dispersion. The excellent agreement between the experimental data and the cumulative peak fitting using Lorentzian functions confirms the reliability of the spectral deconvolution. The presence of all expected phonon modes, along with slight modifications due to Te incorporation, validates the formation of a high-quality WSe_1.95_Te_0.05_ alloy with minimal phase separation. This spectral analysis provides deeper insights into the vibrational dynamics, alloy disorder, and the impact of Te doping on the phononic properties of WSe_2_. Generally, in the case of WSe_2_, the two peaks of lower frequency (E_2g_^1^) and higher frequency (A_1g_) can be observed at 250 and 260 cm^–1^, respectively.^20,21^ The slight shifting of these peaks confirms the appearance of Te in the WSe_1.95_Te_0.05_ single crystal. These results confirm the material’s structural integrity and well-defined vibrational modes.

**Figure 1:**
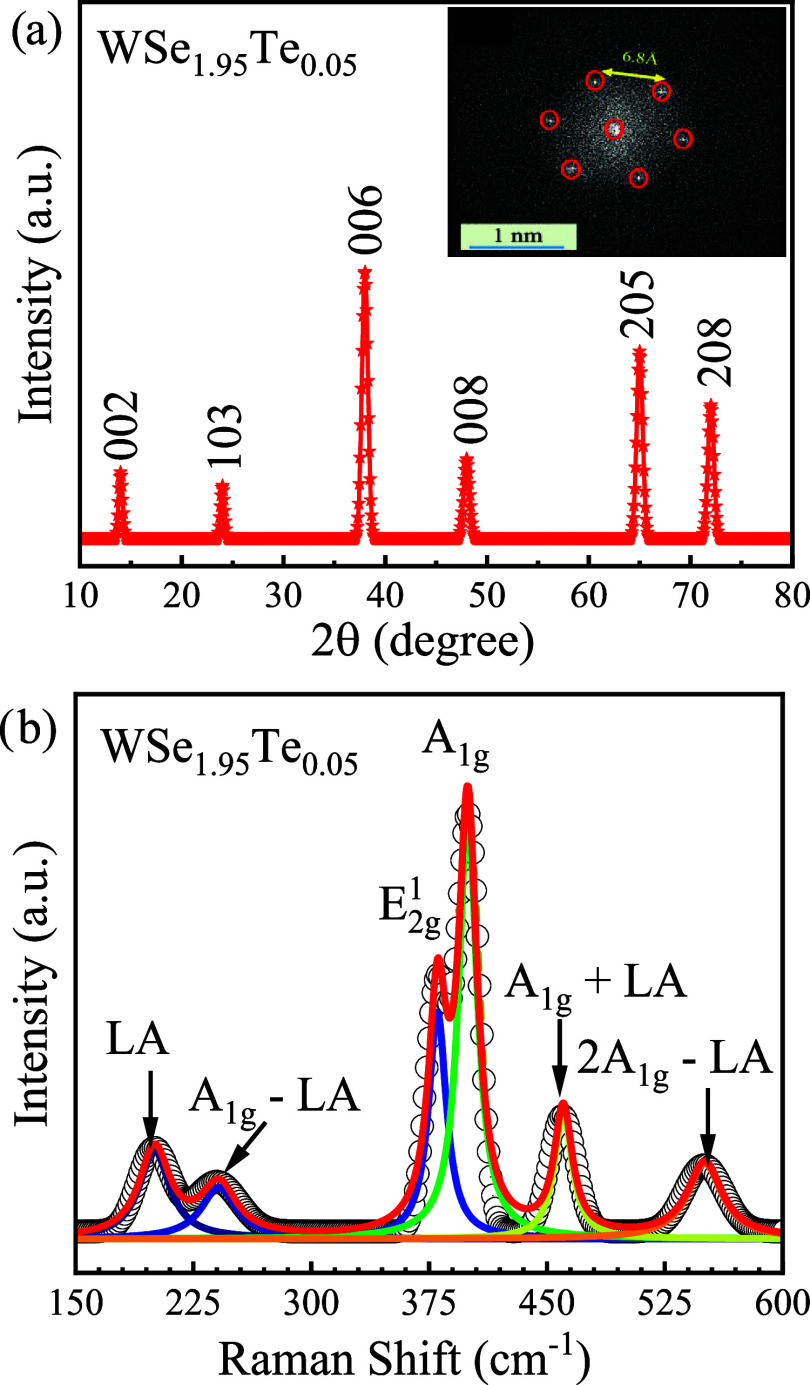
(a). Single-crystal XRD of WSe_1.95_Te_0.05_ with full width at half-height (fwhm) of the peak 002 is 0.14° with well-indexed peaks along hkl, and in the inset selected area Electron Diffraction (SAED) pattern of WSe_2_ with interatomic separation of 6.8 Å elucidating the hexagonal structure (b) Lorentz fitted deconvoluted Raman vibrational spectrum elucidating the influenced Raman active phonon modes due to Te doping in WSe_2_.

The WSe_1.95_Te_0.05_ nanosheets were mechanically exfoliated from bulk crystals using dicing tape and transferred onto SiO_2_ (300 nm)/n-Si templates with prepatterned Ti/Au electrodes. Platinum (Pt) contacts were then deposited onto the nanosheets using focused-ion beam (FIB) lithography. Two nanosheets, labeled as S1 for Sample-1 and S2 for Sample-2, were prepared for photocurrent measurements. The scanning electron microscopy (SEM) images, as shown in Figure 2a,b, provide a detailed cross-sectional view of the WSe_1.95_Te_0.05_ heterostructure integrated with platinum (Pt) contacts, illustrating the structural integrity, thickness, and dimensional characteristics of the fabricated device. These images were obtained using FIB milling, which allows precise cross-sectional imaging by selectively removing material while preserving the heterostructure’s interfaces. In Figure 2a, the WSe_1.95_Te_0.05_ flake exhibits well-defined lateral dimensions of 1.236 × 1.469 μm, confirming successful exfoliation and transfer of the thin layer. The vertical distance measurements at the top inset indicate a thickness range of 45.771 to 47.688 nm, suggesting slight variations in the exfoliated flake’s thickness due to the inherent surface roughness or nonuniformity in the deposition process. Similarly, in Figure 2b, another section of the sample displays a slightly larger exfoliated flake with lateral dimensions of 2.136 × 2.171 μm, with thickness measurements ranging from 43.191 to 45.085 nm. The Pt layers serve both as protective caps and as electrical contacts, ensuring minimal degradation of the WSe_1.95_Te_0.05_ layer and facilitating electrical transport measurements. The sharp and well-preserved interfaces observed between WSe_1.95_Te_0.05_ and Pt confirm the high quality of the heterostructure with no visible defects or voids, ensuring effective charge transport across the junction. These structural characterizations confirm that WSe_1.95_Te_0.05_ is successfully exfoliated into thin layers with thicknesses in the ∼45 nm range, making it highly suitable for nanoelectronics and optoelectronic applications. The uniformity in thickness and seamless integration with Pt electrodes further highlight the potential of WSe_1.95_Te_0.05_ for device fabrication and transport studies in low-dimensional semiconductor systems.

**Figure 2:**
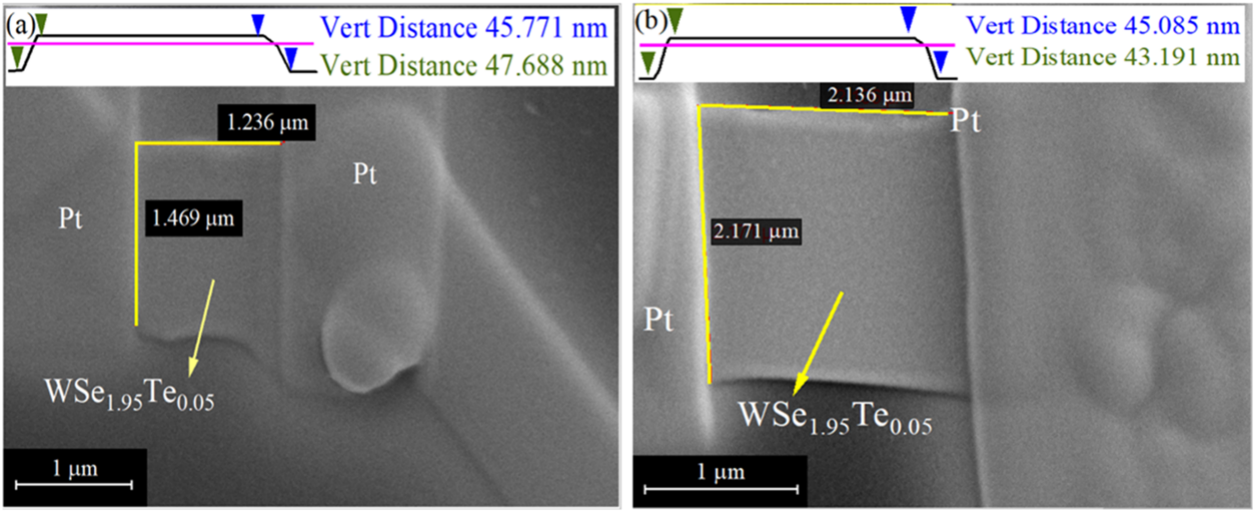
SEM image at 1 μm scale of WSe_1.95_Te_0.05_ of two different nanosheets fabricated by Pt electrodes: (a) initial structure and (b) more refined with squared-off structure. In the upper inset, the AFM with their respective vert distance.

For the study of photocurrent with respect to applied voltage, the *I–V* characteristics have been plotted at 532 nm wavelength, and the same is shown in Figure 3a,b for two samples of pristine and half-years exposed. The photocurrent (*I*_ph_) vs time is measured under different applied bias voltages ranging from 0.1 to 0.5 V. The measurements compare the device performance at two time points: immediately after fabrication (0-day measurement) and after half a year (six-month measurement). This analysis is crucial in assessing the material’s environmental durability and long-term stability for optoelectronic applications. In Figure 4a, the initial photoresponse measured on day 0 exhibits well-defined and steady-state photocurrent generation when the device is exposed to illumination. The photocurrent systematically increases with applied bias voltage, indicating efficient charge carrier separation and transport in the WSe_1.95_Te_0.05_ material. The stepwise increment of *I*_ph_ with increasing voltage (from 0.1 to 0.5 V) suggests that the material exhibits a voltage-dependent photocurrent response, characteristic of a semiconductor with a well-defined band structure. However, after six months, a noticeable reduction in the photocurrent magnitude is observed across all applied bias voltages, suggesting a slight degradation in the material’s optoelectronic properties over time. Despite this, the overall trend and behavior of the device remain consistent, indicating that the material retains a substantial portion of its original functionality. While photocurrent measurements are widely used to infer long-term device reliability, many layered materials undergo surface-limited oxidation, which is often self-terminating and affects only the topmost layers, leaving the bulk properties largely intact.^23,24^ This type of oxidation can mildly reduce the surface conductivity or photoresponse yet does not compromise the core functionality of optoelectronic devices.^25,26^ Similarly, Figure 3b demonstrates a comparable trend, where the photocurrent initially shows a strong, bias-dependent response on day 0, followed by a minor degradation after six months. The shape of the transient response remains unchanged, confirming that the switching dynamics and response time of the device are not significantly affected by prolonged environmental exposure. This suggests that while the magnitude of the photocurrent decreases slightly, the fundamental charge transport and recombination mechanisms remain largely intact. The consistency in the rise and decay times of the photocurrent further indicates that no significant trap-state formation or defect-induced recombination centers have developed over this period, which is a critical factor in determining the long-term usability of the material. The observed degradation in photocurrent over time can be attributed to several potential factors, including environmental adsorption (such as oxygen and moisture), oxidation of the material’s surface, or structural modifications due to prolonged exposure to ambient conditions. In particular, Te incorporation in WSe_2_ is known to influence stability, and the slight photocurrent reduction suggests that the presence of Te may introduce additional defect states or alter the intrinsic carrier lifetime upon prolonged exposure. However, it is important to note that the overall stability remains relatively high, as the device retains a significant fraction of its initial photocurrent response even after six months. It has been observed that the photocurrents remain unpretentious under ambient conditions for over six months for both samples. Generally, the observed time of rise and decay of photocurrent is 7 s for 2D materials, including TMDs. For the WSe_1.95_Te_0.05_, we have observed the same rise and decay time, which elucidates the stable and reliable photoresponse. The ON/OFF ratio of photocurrent is found to be around 200 under 0.5 V applied voltage with 13 nA measured dark current. The higher ratio value of ON/OFF suggests the nature of light sensitivity with competent shifting between conducting and insulating states. This aligns with earlier studies where 2D TMD-based photodetectors maintained performance over long durations despite superficial environmental changes.^27,28^ These results highlight the intrinsic environmental durability of WSe_1.95_Te_0.05_ and suggest that Te-substituted WSe_2_ alloys could serve as promising candidates for long-term optoelectronic applications. Furthermore, Te doping in WSe_2_ may passivate active surface sites, thereby enhancing resistance to oxidation and contributing to overall device robustness.^29,30^ Recent reports also emphasize that Te substitution tunes the charge transport, suppresses trap states, and improves ambient resilience in optoelectronic applications.^31,32^ The observed preservation of response time and switching symmetry over six months strongly supports these conclusions. Additionally, the studies were conducted on two nanosheets, with similar results, as shown in Figure 3c,d. The photocurrent for both samples exhibits a linear dependence on the applied voltage, further confirming the metallic behavior of the WSe_1.95_Te_0.05_ system.^33–36^ It is proposed that the as-grown single-crystal WSe_1.95_Te_0.05_ behaves as a semiconductor with an optical bandgap of 1.35 eV. It is well-established that conventional Ti/Au electrodes can form nonohmic contacts, creating Schottky barriers between Pt and WSe_1.95_Te_0.05_, with a bandgap of 5.1 eV at the interface with 2D TMDs.^37,38^ These nonohmic contacts introduce potential barriers that can lead to electron–hole annihilation, negatively affecting the intrinsic photocurrent response. The resistivity measurements of the two nanosheets revealed values of 1.45 × 10^–4^ Ω-m for S1 and 8.72 × 10^–5^ Ω-m for S2. Despite having similar dimensions, the difference in resistivity is likely due to defects introduced during the exfoliation and fabrication processes as well as contributions from the probing wires and electrodes. Remarkably, after six months of ambient exposure, the resistivity remained unchanged, demonstrating the oxidation-resistant carrier transport properties of WSe_1.95_Te_0.05_. A similar behavior is observed in other 2D materials.^39–42^ This stability is crucial for its potential use in reliable and long-lasting electronic applications. Furthermore, under reverse bias, the photocurrent response becomes less sensitive to the applied voltage and remains relatively low. This behavior suggests that the use of Pt contacts helps minimize the influence of the contact barriers, thereby optimizing the intrinsic photoresponse of WSe_1.95_Te_0.05_.^43,44^ By reducing interface-related losses, Pt electrodes contribute to improving the overall efficiency of the material, making it more suitable for optoelectronic applications. This underscores the crucial role of electrode material selection in enhancing the performance of 2D semiconductors.

**Figure 3:**
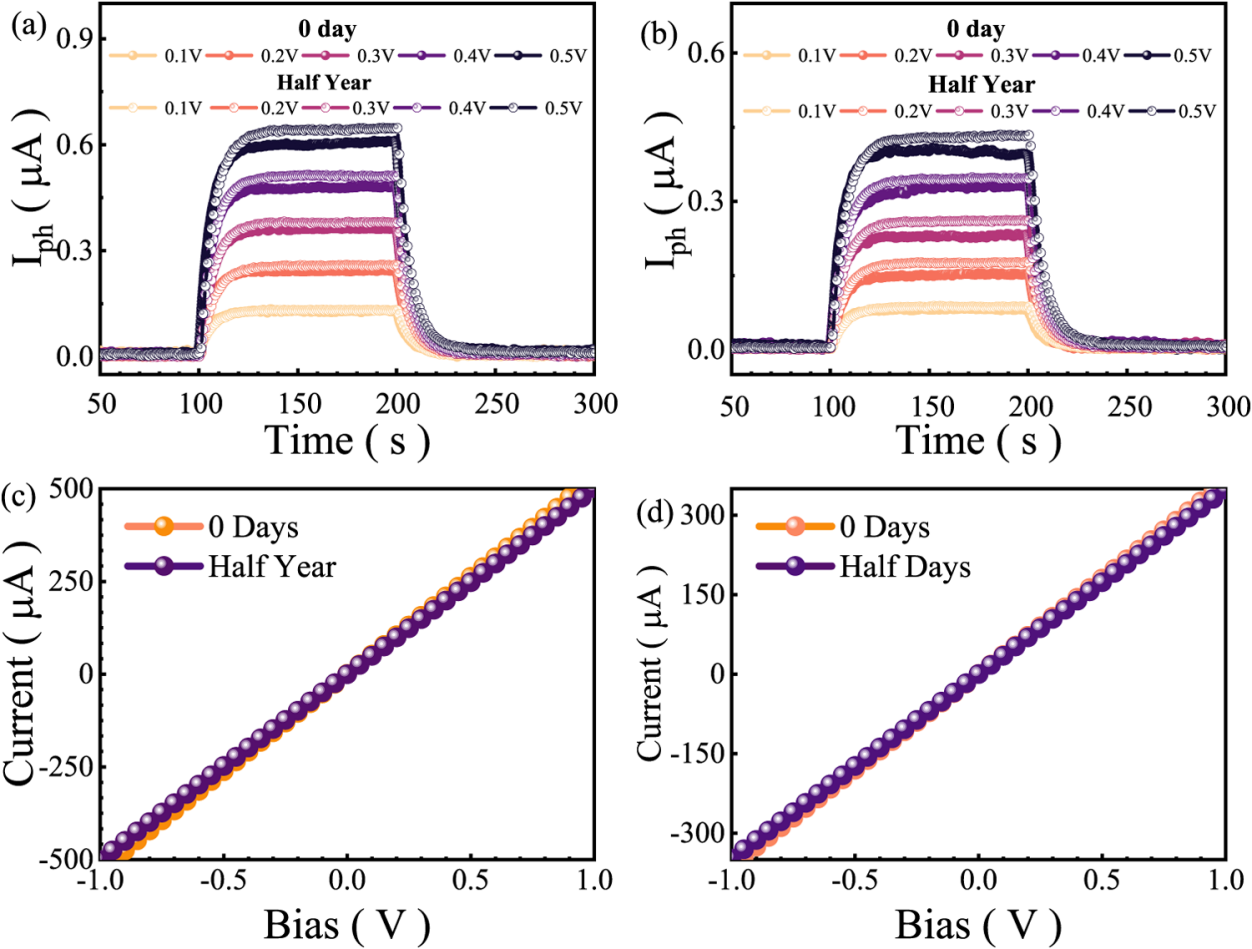
(a) and (b) Photocurrent response (*I*_ph_) vs time at different applied voltages (0.1–0.5 V) elucidating that the photocurrent remains almost unchanged after half a year, indicating good stability of the material/device. This exhibits a similar response time (rise and decay characteristics) even after long-term storage, with no significant degradation in photocurrent amplitude observed, (c) and (d) the *I–V* curves (current vs bias voltage) for the device at 0 days and after half a year. The near-identical linear behavior suggests that the electrical contacts remain stable, with no significant resistance change over time. The material does not exhibit major degradation or defect formation, proving its long-term reliability.

**Figure 4:**
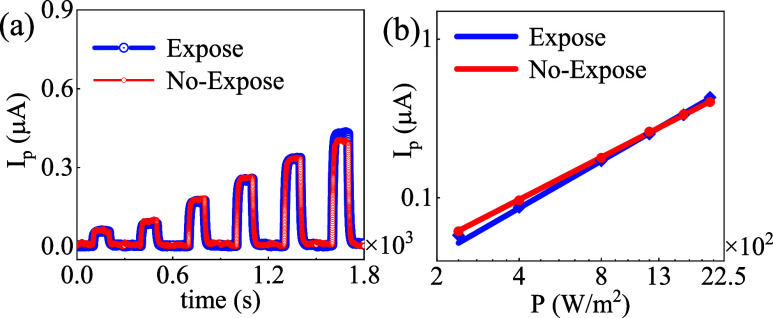
(a) Elucidating the variation of *I*_ph_ (photoinduced current) with time for two conditions, “Expose” and “No-Expose”. The “Expose” condition results in higher peaks compared to “No-Expose” and has a higher response, and the current exhibits a step-like pattern, increasing periodically over time. (b) Shows the relationship between *I*_p_ and P. The “Expose” condition results in slightly higher current values than “No-Expose”. The trend appears to be linear on a log–log scale, indicating a power law dependence of *I*_p_ on P.

The photocurrent as a function of light power intensity was investigated, with the results shown in Figure 4a. These figures illustrate the photocurrent response for both pristine and half-year-exposed WSe_1.95_Te_0.05_ nanosheets at a wavelength of 532 nm. The study reveals a significance enhancement of photocurrent on increasing the power intensity for both pristine and half-year exposed. To further analyze the relationship, Figure 4b presents the power-intensity-dependent photocurrent on a double-logarithmic scale, revealing a linear correlation between the photocurrent and light power intensity. This behavior confirms that the photocurrent is directly proportional to the incident light intensity. The photocurrent relationship is modeled by *I*_P_ = AP^β^, where β*(x*) reflects the underlying carrier transport mechanism. The fitting results indicate β­(*x*) = 1.0 for Sample-S1 and β­(*x*) = 0.98 for Sample-S2. A β value close to 1 implies that the photocurrent is primarily governed by direct photogeneration with negligible influence from trap-assisted recombination, suggesting low defect density and superior crystal quality in the alloyed material.^45,46^ In low-dimensional materials, the slight deviation in β­(*x*) from 1 implies attribution to photogating effects induced by deep-level traps and carrier recombination delays.^47^ However, there are no complex photon-carrier coupling mechanisms in our system, which reflect the minimal defect trapping, reaffirming the simple linear dependence between the photocurrent and light intensity. Such behavior has also been reported in Te-alloyed TMDs, where Te substitution reduces chalcogen vacancies and passivates trap states, enhancing optoelectronic performance and stability.^48,49^ The photocurrent responsivity, RRR, is given by *R ∼ I*_ph_/*I* represents the photocurrent, and *I* is the light power intensity. For sample (S2), the sublinear relationship indicates that the responsivity decreases with increasing intensity, following the power law *R ∼ I* ^0.02^, again confirming that charge trapping and photogating are not dominant. The reduced slope in responsivity at higher intensities may originate from mild saturation effects or weak surface adsorbate interactions. Unlike many reports in the literature, our results show that the photocurrent in WSe_1.95_Te_0.05_ exhibits a minimal dependence on light intensity. This weak power dependence resembles the hole trapping effect in *n*-type semiconductors and may be associated with carrier trapping phenomena. Table 1 presents the photocurrent responsivity values of various 2D TMDs. Two mechanisms are responsible for the observed behavior. The first mechanism involves the physical adsorption of molecules on the sample surface at lower pressures. This process allows more photons to penetrate the material, thereby generating a higher number of carriers. The second mechanism is linked to chemical adsorption–desorption, where ambient air molecules facilitate the recombination of photon-excited electron–hole pairs. Specifically, oxygen or water molecules can capture the photon-generated electrons, chemically adsorbing onto the surface of WSe_1.95_Te_0.05_ while simultaneously trapping the photon-generated holes and desorbing from the surface. This chemical adsorption–desorption cycle occurs more rapidly than the typical electron relaxation processes, especially in systems with a large surface-to-volume ratio. Consequently, this effect reduces the number of photon-generated electron–hole pairs, resulting in a lower photocurrent at higher pressures. It is expected that higher pressures will increase the number of oxygen molecules or water molecules adsorbed on the surface.

**Table 1:** Compilation of Reported Photocurrent Responsivity Values in 2D TMDs

**2D-material**	**material type**	**responsivity (AW** ^**–1**^ **)**	**wavelength (nm)**	**bias (V)**	**references**
WSe_1.95_Te_0.05_	(S1, pristine)	65	532	0.5	this work
	(S2, pristine)	108			
	(S1, half-year)	69			
	(S2, half-year)	115			
GaSe	film	2.8	254	5	^61^
GaS		4.2		2	^62^
MoS_2_	bulk	4.2 × 10^–4^	670	1	^63^
		6	532	5	^64^
		0.57	532	10	^65^
		0.12	633	1	^29^
		4.1	655	5	^66^
MoS_2_	nanoflake	30	532	0.1	^67^
		0.06	365	2	^68^
MoS_2_	APTES-doped	56.5	655	5	^69^
	OTS-doped	0.36			
WSe_2_	APTES-doped	20			
	OTS-doped	0.59			
	pure	364			
WS_2_	film	0.7	635	9	^70^
	few layers	53.3	365	5	^71^
	pure	0.99 × 10^–6^	532	10	^72^
Nb-doped WSe_2_	bulk	1.2	652	10	^43^
	few layers	3.5		5	^73^
WSe_2_	few layers	0.15	532	1	^74^
	film	0.92	635	10	^75^
Mo_0.5_W_0.5_S_2_	polycrystal film	5.8	635	2.2	^76^
In_2_Se_3_	nanosheet	395	300	5	^77^
		110	400		
		59	500		
InSe	layers	0.1	532		^78^
		12.3	450	10	^79^
NbSe_2_	nanoflake	2.3	532	0.1	^80^
		3.8	808	0.1	
SnS_2_	bulk	1100	530	3	

The optoelectronic performance of WSe_1.95_Te_0.05_ was systematically investigated under different pressure conditions, focusing on its photocurrent (*I*_ph_) and responsivity (*R*), as shown in Figure 5a,b. The time-dependent photocurrent response in Figure 5a reveals a significant dependence on pressure, with *I*_ph_ increasing as the pressure decreases. This trend suggests that at lower pressures, charge carrier recombination is suppressed, leading to an enhanced photocurrent. The reduced molecular adsorption of atmospheric gases, such as oxygen and water vapor, minimizes charge trapping effects, thereby improving carrier transport within the material. Moreover, the rise and decay dynamics of *I*_ph_ indicate an efficient charge generation and separation process, with lower pressures facilitating longer carrier lifetimes due to decreased nonradiative recombination pathways. Comparing the two samples, Sample-2 consistently exhibits a higher photocurrent than Sample-1 across all pressure conditions, indicating superior charge carrier mobility, reduced defect states, or improved photoresponsivity, possibly due to differences in crystal quality, doping uniformity, or fabrication processes.^50^ Figure 5b presents the variation of the responsivity (*R*) as a function of pressure on a logarithmic scale, further emphasizing the strong pressure dependence of WSe_1.95_Te_0.05_. The decreasing trend of *R* with increasing pressure highlights that lower pressure conditions enhance the photodetection efficiency of the material. This behavior can be attributed to reduced gas interactions at lower pressures, which decrease the probability of charge carrier trapping by adsorbed molecules. The power law dependence observed in *R* versus pressure suggests that surface states and external environmental factors play a crucial role in modulating the optoelectronic properties of WSe_1.95_Te_0.05_. The pressure dependence of *R* can be expressed as *R/pm*, where *p* represents pressure and m is a fitting parameter that depends on the ambient gas. The fitting results show *m* = −0.11 for sample (S1) and *m* = −0.09 for sample (S2), suggesting that m is influenced by the presence of physically adsorbed molecules.^51^ Notably, Sample-2 consistently demonstrates a higher responsivity than Sample-1, reinforcing its superior photodetection capabilities. The enhanced performance of Sample-2 suggests the potential influence of material synthesis parameters, defect engineering, or doping effects on the overall optoelectronic response. These findings suggest that WSe_1.95_Te_0.05_ is highly sensitive to pressure variations, making it an excellent candidate for low-pressure photodetection applications. The ability of this material to exhibit enhanced photocurrent and responsivity at reduced pressures makes it suitable for applications in vacuum-based sensors, space instrumentation, and environmental monitoring. The observed pressure dependence also highlights the importance of optimizing synthesis techniques to minimize defects and maximize carrier mobility, further improving the performance of WSe_1.95_Te_0.05_-based photodetectors. These results provide valuable insights into the tunability of TMD-based materials for advanced optoelectronic applications, emphasizing the role of environmental conditions in dictating material performance.

**Figure 5:**
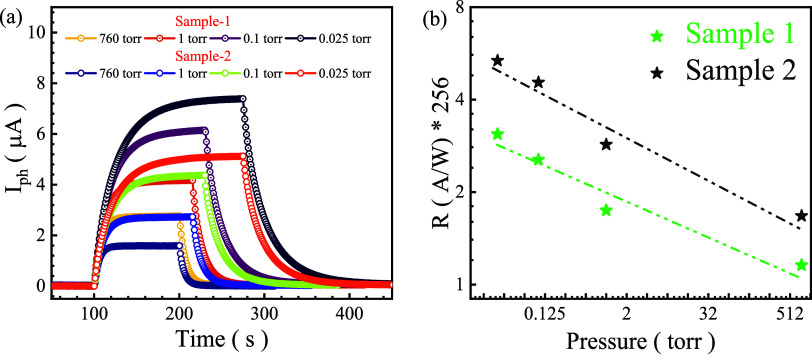
(a) Elucidating the variation of photocurrent (*I*_ph_) over time for two different samples (Sample-1 and Sample-2) under different pressures (760, 1, 0.1, and 0.025 Torr). Both samples exhibit an increase in *I*_ph_ over time, reaching a saturation level. Sample-2 shows a higher photocurrent than Sample-1 under the same pressure conditions, with a higher photocurrent for lower pressure conditions. (b) Responsivity (*R*) as a function of pressure for both samples. Sample-2 exhibits consistently higher responsivity than Sample-1 across all pressure values. The data follows a decreasing trend, suggesting an inverse relationship between responsivity and pressure as the responsivity decreases on increasing the pressure for both samples.

Figure 6 presents the dependence of responsivity *R* (A/W) on incident power density (*P*, W/m^2^) for WSe_1.95_Te_0.05_ under illumination at three different wavelengths: 405, 532, and 808 nm. A distinct decreasing trend in *R* with increasing *P* is observed across all wavelengths, indicating a power-dependent saturation mechanism.^52^ Initially, at low power densities, the responsivity is high, suggesting efficient photocarrier generation and collection. However, as the power density increases, a decline in *R* is observed, which can be attributed to several factors, such as trap-state filling, increased nonradiative recombination, and possible thermal effects that lead to a reduction in carrier lifetime or mobility.^53^ Among the three wavelengths, the highest responsivity is recorded at 405 nm, implying stronger absorption in the ultraviolet–visible region due to the high photon energy exciting electrons efficiently across the bandgap. The relatively high *R* at this wavelength suggests that the material exhibits a high absorption coefficient in the UV-blue region, which is beneficial for photodetection in this range. For the 532 nm wavelength, the responsivity is lower than that at 405 nm but still follows a similar decreasing trend with the power density. This indicates that while the material can absorb green light, the absorption efficiency is reduced compared to that of the shorter wavelength, possibly due to a lower absorption coefficient at this energy range. Crucially, the device still demonstrates a discernible photoresponse at 808 nm, even though pristine WSe_2_ typically exhibits a direct bandgap absorption edge near 750 nm.^54,55^ This NIR response indirectly confirms that Te substitution at Se sites has effectively narrowed the bandgap of WSe_2_, shifting the absorption edge to longer wavelengths. Such bandgap reduction is a known outcome of isoelectronic Te alloying, which introduces lattice strain and modifies the electronic structure by lowering the conduction band minimum. At 808 nm, the responsivity is the lowest among the three wavelengths, suggesting weak absorption in the near-infrared region. This behavior is likely due to the band structure of WSe_1.95_Te_0.05_, where the incident photon energy at 808 nm (corresponding to ∼1.53 eV) may be close to or lower than the bandgap energy, leading to inefficient electron–hole pair generation. The declining trend of *R* with an increase in *P* at 808 nm further supports the idea of saturation effects dominating the charge carrier dynamics. Another key observation is that while responsivity decreases with power density for all wavelengths, the rate of decline varies. At 808 nm, the drop in *R* is more pronounced, indicating a stronger saturation effect, likely due to the combination of weaker absorption and increased carrier recombination. At 532 and 405 nm, the reduction in responsivity is more gradual, with 405 nm showing a slight recovery at the highest power density, suggesting possible nonlinear absorption mechanisms or a photoconductive gain effect at high excitation levels. Overall, these results highlight the wavelength-dependent optoelectronic response of WSe_1.95_Te_0.05_, demonstrating its strong photoresponse in the visible range while exhibiting limited absorption in the near-infrared region. The power-dependent responsivity behavior suggests the presence of nonlinear carrier dynamics, making this material a potential candidate for tunable photodetectors, particularly for applications requiring high sensitivity in the UV–visible region.

**Figure 6:**
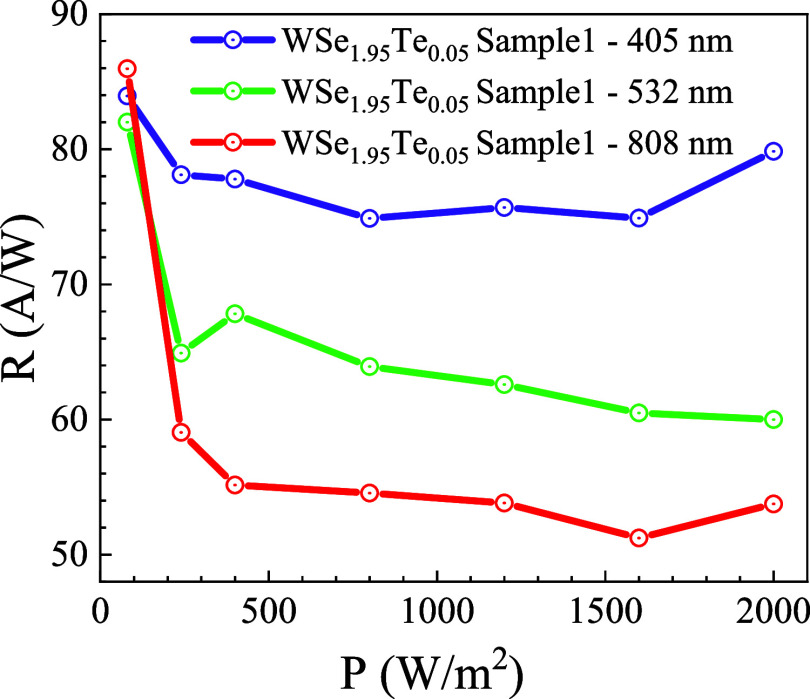
Elucidating the responsivity (*R*) as a power intensity function at three different wavelengths. The responsivity (*R*) of WSe_1.95_Te_0.05_ decreases sharply with increasing power density (*P*) before stabilizing, with the highest responsivity observed at 405 nm, followed by 532 nm, and the lowest at 808 nm, indicating a wavelength-dependent photoresponse likely due to varying absorption efficiency and carrier recombination effects.

Figure 7 shows the XPS analysis of WSe_1.95_Te_0.05_, which provides valuable insights into the electronic structure, bonding characteristics, and chemical environment upon Te incorporation. In the W 4f core-level spectrum, as shown in Figure 7a, the characteristic spin–orbit doublet corresponding to W 4f_7/2_ and W 4f_5/2_ exhibits slight binding energy shifts compared to pristine WSe_2_, indicating modifications in the tungsten oxidation state and local electron density.^56^ This shift suggests that Te substitution affects the charge distribution, potentially altering the hybridization between W and chalcogen sites. Similarly, the Se 3d spectrum, as shown in Figure 7b, reveals multiple deconvoluted peaks, signifying variations in the chemical states of selenium. These shifts may arise due to a change in the local bonding environment upon Te incorporation, as tellurium has a larger atomic radius and different electronegativity compared to selenium, influencing the W–Se and W–Te interactions.^57,58^ The presence of Te is confirmed by the Te 3d spectrum, as shown in Figure 7c, which displays two distinct peaks corresponding to Te 3d_5/2_ and Te 3d_3/2_. Notably, additional features in the spectrum correspond to TeO_2_, suggesting partial oxidation of Te, possibly due to environmental exposure. This oxidation effect indicates that Te is more susceptible to chemical interactions, which may impact the long-term stability of the material. The introduction of Te is also expected to induce local lattice distortions as Te atoms have a larger atomic radius (∼140 pm) than Se (∼120 pm). This size mismatch can generate strain in the crystal lattice, affecting the structural stability and modifying the electronic band structure. Furthermore, the substitution of Te into the WSe_2_ matrix is likely to influence the bandgap due to the weaker W–Te bond compared to W–Se.^59,60^ A reduction in the bandgap is expected, which can enhance electrical conductivity and improve the material’s suitability for optoelectronic applications. The Te doping may also introduce additional defect states, which could impact charge carrier recombination and transport mechanisms.

**Figure 7:**
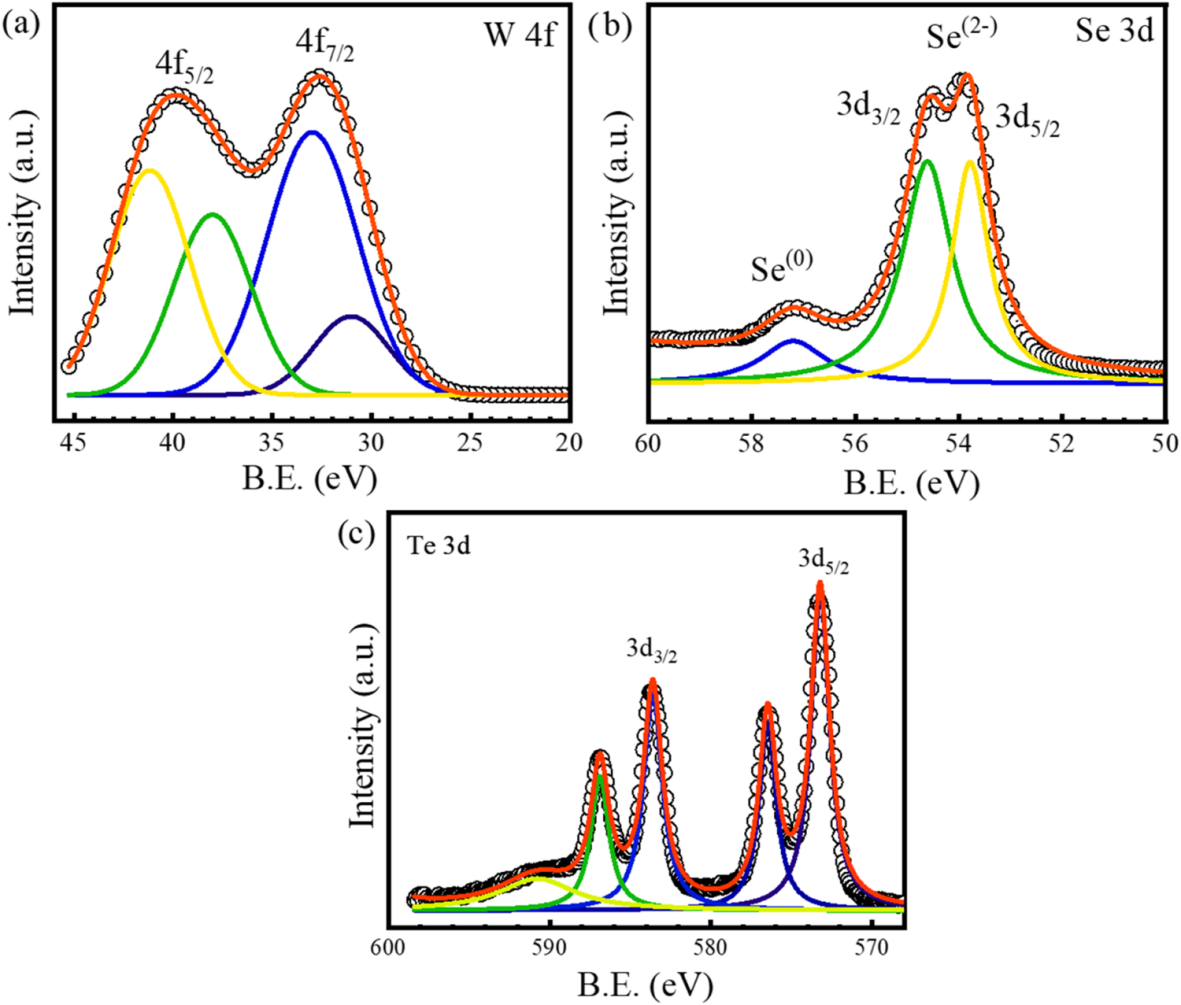
X-ray photoelectron spectroscopy (XPS) analysis of WSe_1.95_Te_0.05_, highlighting the chemical states and electronic structure modifications due to Te doping. The deconvoluted spectra for the (a) W 4f, (b) Se 3d, and (c) Te 3d core levels demonstrate shifts in binding energies, confirming the successful incorporation of Te into the WSe_2_ lattice. Additionally, the presence of TeO_2_ peaks indicates partial oxidation of Te, which influences the surface chemistry.

## Conclusions

The WSe_1.95_Te_0.05_ semimetal nanosheets demonstrate remarkable electrical and optical properties, making them highly suitable for optoelectronic applications. A key advantage of this material is its ability to form stable ohmic contacts, ensuring efficient charge carrier transport without significant energy barriers at the electrode interface. This stable contact formation contributes to the material’s photoresponsivity, measured at 108 A/W without any surface treatment, highlighting its intrinsic optoelectronic efficiency. The XPS analysis confirms successful Te incorporation, leading to electronic structure modifications, lattice strain, and partial oxidation effects. Notably, the device maintains an impressive responsivity of 115 A/W even after six months, indicating exceptional long-term stability and durability under ambient conditions, which is a crucial requirement for practical device applications. Additionally, WSe_1.95_Te_0.05_ exhibits power-independent photocurrent behavior, meaning that its photocurrent response does not significantly vary with incident power density. This stability suggests the absence of complex photon-carrier coupling mechanisms, such as trap-assisted recombination or nonlinear absorption effects, which are often observed in other materials. The material’s ability to maintain consistent performance over extended periods without degradation further enhances its potential for real-world applications. These findings highlight the intrinsic qualities of WSe_1.95_Te_0.05_ and position it as a strong candidate for next-generation optoelectronic devices, particularly in high-performance photodetectors and photosensors requiring high sensitivity, stability, and efficiency across a broad operational range.
